# Strive for excellence: unboxing the antecedents of art design students’ creativity in the learning process in China

**DOI:** 10.3389/fpsyg.2026.1785090

**Published:** 2026-03-11

**Authors:** Jin Jianhao, Veronica Ng, Lim Chong Hin, Mohamed Rizal Mohamed, Md Sazzad Hossain

**Affiliations:** 1 School of Fine Arts and Design, Heze University, Heze, China; 2 School of Architecture, Building and Design, Taylor's University, Subang Jaya, Malaysia; 3 Faculty of Arts and Social Sciences, Sunway University, Subang Jaya, Malaysia; 4 Faculty of Built Environment, Universiti Teknologi MARA, Shah Alam, Malaysia; 5 College of Tourism and Hospitality, University of Tabuk, Tabuk, Saudi Arabia

**Keywords:** creativity in the learning process, disciplinary value, emotional management, learning environment, learning style, personality characteristics, reflective practice, self-efficacy

## Abstract

**Objectives:**

The study aims to examine how personality characteristics, disciplinary values, self-efficacy, emotional management, and learning style affect student creativity during the learning process. Later, determine the mediating role of reflective practice and the moderating role of the learning environment.

**Methods:**

A total of 402 final-year Art Design undergraduates from Shandong province in mainland China responded to the questions.

**Results:**

The findings indicate that students’ personality characteristics, self-efficacy, disciplinary values, and emotional management significantly affect reflective practice. Moreover, reflective practice substantially played a role in mediation. Surprisingly, learning style does not significantly affect reflective practice or creativity during learning. The learning environment was actively moderated. Finally, reflective practice and student creativity in the learning process are significantly related.

**Conclusion:**

This study elucidated the learning processes of Chinese Art Design students’ creativity. It is anticipated to offer a novel foundation and viewpoint for improving Art Design education.

## Introduction

In the fourth industrial revolution, creativity has become a core competency for innovation in the higher education sector (McInerney, 2023; Guaman-Quintanilla et al., 2023). It is an indispensable skill for undergraduates to succeed in the increasingly complex, uncertain, and changing world (Thornhill-Miller et al., 2023). The importance of creativity has been a recent focus of higher education reforms in Asian and Chinese societies (Saris, 2020). The Chinese State Council (CSC) has stated that creative talent is the core of creative design and a fundamental pillar for improving the country’s soft power. Given the scale and the catalogue of Art Design (A&D), it is hard to deny that it should be among the leading fields in developing creative design talent (Wang and Liu, 2022).

China has gradually become the most significant country in global design education. However, the importance of A&D students’ personal influence and cognitive learning process has long been ignored in the education literature (Sarwandianto et al., 2020). Besides, there are apparent gaps between Chinese and Western A&D students in creative capabilities, originality, design thinking, and practical experience during their learning (Huang et al., 2020; Saris, 2020). Moreover, in some surveys of graduates (Liu and Nah, 2020), 50% believe that only a portion of what is taught in school is suitable for practical design work. Other aspects, such as problem-solving, cooperation, emotional management skills, and strategic learning, are lower than expected. This problem has negatively impacted students’ professional ability and future competitiveness, as well as the implementation of the national talent development strategy (Karwowski et al., 2020). Thus, understanding how A&D students’ creativity can be stimulated by exploring the factors that influence it is crucial.

Many researchers have recently advocated for a comprehensive study of self-regulated learning (SRL) in A&D education (Greene et al., 2019). Recent studies have proved that students’ creativity can be fostered and enhanced when they possess SRL abilities (Munahefi et al., 2022). Evidence shows that combining SRL and design could bring out the best while motivating students’ achievement and fostering creativity in design pedagogy (Akdeniz and Turan, 2022; Powers, 2016). However, little is known about how A&D students learn to be creative in Chinese institutions, and student-centred studies that address the creative process are primarily absent from educational literature (Orr et al., 2014). Therefore, this study developed a framework to analyse factors influencing student creativity in the learning process, based on the idea that creativity can be influenced by the SRL theory’s personal, behavioural, and environmental aspects.

Furthermore, reflective practice has been considered a critical behaviour and is vital in predicting individual learning outcomes and performance (Kirkman and Brownhill, 2020; Sukarso et al., 2019). It is regarded as a behavioural factor that guides the integration of complex, difficult creative tasks and the identification of specific strategies for generating and refining creative ideas (Alt and Raichel, 2020). Additionally, critical attributes of the learning environment have significantly affected students’ creativity and complex problem-solving capabilities (Fan and Cai, 2022). Although several studies have mentioned these variables and identified their relationships in different fields, none have considered them simultaneously, especially in the Chinese context. To address this gap, this study has integrated essential factors that enhance students’ creativity in the learning process (CILP), making a unique contribution to the A&D discipline. Given the limited prior A&D studies on creativity from an SRL standpoint, this study addresses the critical role of students’ self-regulated learning in developing creativity.

## Theoretical underpinning

Creativity development in the design discipline is highly dependent on domain-relevant skills (Emami et al., 2023), such as visual imagery (Palmiero et al., 2015; Friedlander et al., 2022), tolerance of ambiguity (Stoycheva, 2025), compositional logic (Ingold and Hallam, 2021), and user interaction strategies (Cropley, 2015; Kozbelt et al., 2010). These skills not only provide essential resources for idea generation during the divergent thinking stage but also serve as the foundation for turning ideas into feasible, functional, and context-sensitive design solutions during the convergent stage (Sawyer, 2017). The self-regulated learning (SRL) theory is the basis for the proposed model. SRL is a great umbrella covering various learning variables (e.g., self-efficacy, emotion, motivation, cognitive strategies) that have been studied holistically. In SRL, individuals must manage thoughts, emotions, behaviours, and environments to fulfil their academic objectives (Panadero, 2017). Klimova and Pikhart (2022) found that students with higher SRL skills were better able to adapt to the learning environment and overcome challenges. Li et al. (2025) found that students with strong SRL skills can better manage their time, set appropriate goals, and monitor their progress toward those goals.

**Figure 1:**
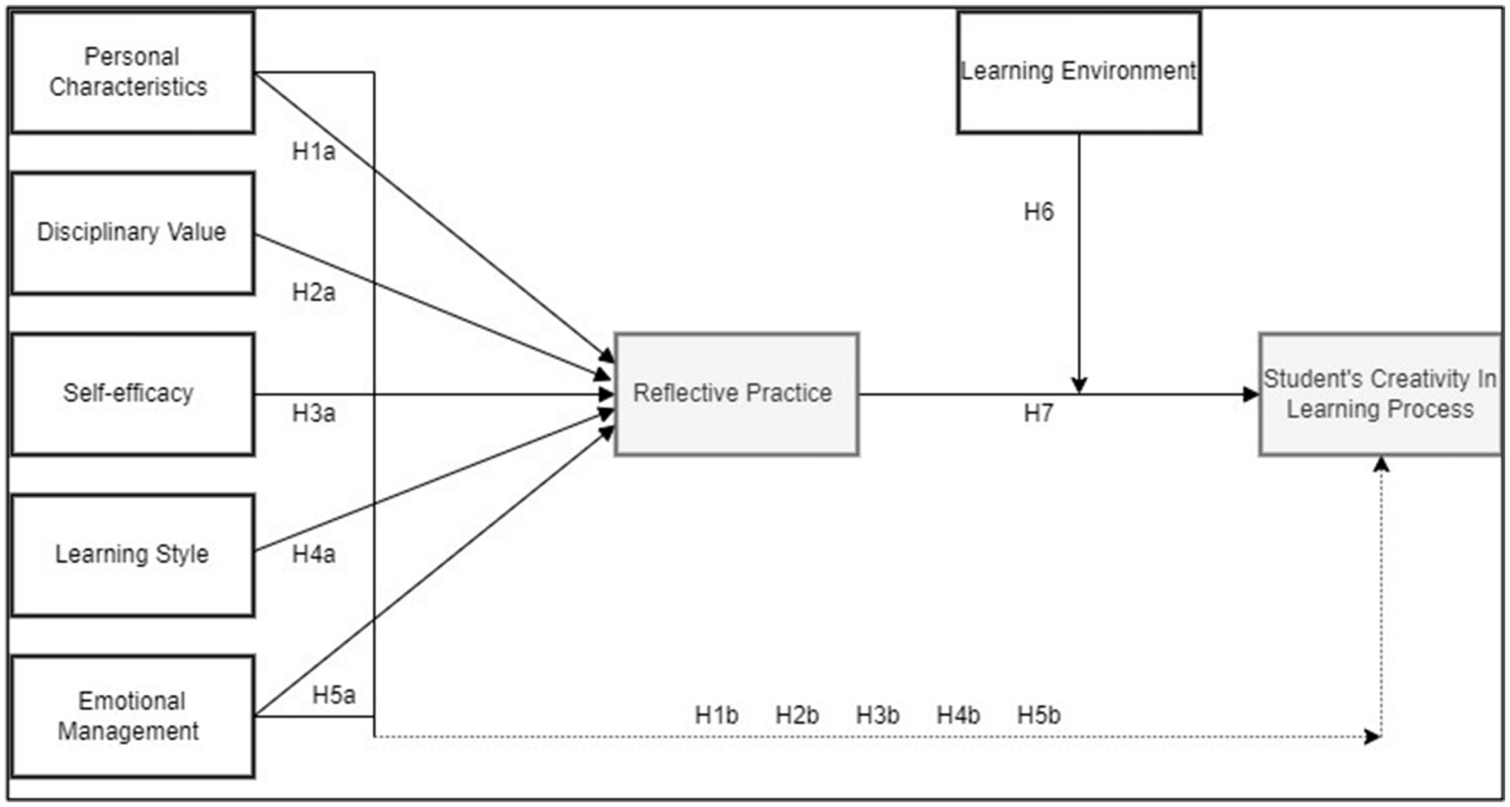
The conceptual research model.

Moreover, scholars have suggested that SRL related to students’ metacognition is associated with creativity and active learning (Zielińska et al., 2023). In design education, students’ initiative, reflection, and creative processes are crucial for developing design creativity (Robson et al., 2020). Recent studies have shown that students’ creativity can be fostered when they possess SRL skills (Munahefi et al., 2022). Studies of learning engagement and creative processes within SRL can inform how these processes are taught and learned in A&D education (Rubenstein et al., 2018). There is also evidence that combining SRL and design can yield the best results while motivating student achievement (Greene et al., 2019). Therefore, embedding creativity research within the SRL framework may help clarify the mechanisms underlying active learning processes and creativity development among A&D undergraduate students (Figure 1).

## Literature review

### Personal characteristics

Drawing on the SRL theory, underlying personality characteristics may be the driving force and source of regularity in information processing (Yao and Li, 2021). These characteristics are then quantified as learning characteristics, as they may influence how people approach and engage in the learning process. Studies from the famous Big Five personality model (Goldberg, 1992) have shown that personality can help prepare individuals for self-reflection, facilitating creative engagement and outcomes. Coan (2019) argued that the ability to engage in self-reflection is closely related to personality traits, as they influence a person’s receptivity to new ideas. Snyder et al. (2021) suggest that people with high openness and flexibility are more likely to engage in self-reflection. Recent research on the relationship between individual differences and language teaching suggests that personality types are essential for learning reflection (Incheh and Tasnimi, 2022).

Certain personality traits can inspire an individual’s innovative performance in design activity (Sosa and Kayrouz, 2020). Some studies found that among the five factors, openness to experience, extraversion, agreeableness, and conscientiousness are positive predictors of creativity (Kenett et al., 2023). Moreover, reflective practice has been found to mediate the relationship between students’ personalities and creative skills in the learning process. Reflective practice cultivates metacognition and the ability to think about one’s thoughts. It allows individuals to use their personality traits to enhance creative thinking (van Diggele et al., 2020). For instance, Kaufman (2018) found that openness predicts self-reported reflection and learning from experience. Russell and Martin (2017) emphasised reflective practice as a continuous process in which learners thoughtfully consider their experiences and practice under the supervision of professionals.

Lutz et al. (2015) underpinned that reflective practice (reflecting on real challenges and in a group setting with the trainer) can enhance students’ creative individuality in difficult communication situations. In line with students’ personalities and openness to experience, creativity is directly predicted, and reflective practice is enhanced. For instance, an individual with high openness might actively seek out diverse experiences and then reflect upon them to extract creative insights. Understanding how certain personalities can lead to high-quality reflective behaviour is valuable for students seeking to foster creative thinking. However, little is known about this relationship in the context of A&D students’ learning processes and their perspectives on personality characteristics.

*H1a:* Students’ personality characteristics significantly influence their reflective practice.

*H1b:* Students’ reflective practice mediates the relationship between students’ personality characteristics and creativity in the learning process.

### Disciplinary values

Disciplinary value correlates positively with SRL strategies and influences students’ learning performance and behavioural outcomes (Patel and Metersky, 2022). This concept underlines that students are more likely to engage actively and invest effort in learning when they perceive a discipline as valuable and relevant to their goals. When students perceive high disciplinary value, they are more likely to reflect deeply as they are intrinsically motivated to succeed in that subject. Phan and Ngu (2014) tested a conceptual model depicting the interrelations among non-cognitive and cognitive learning processes and academic achievement outcomes.

When learners identify with disciplinary values related to their field or task, they are more likely to engage in reflective practice to deepen their understanding and competence. Disciplinary value is a strong predictor of interest, effort, performance, and metacognitive behaviour, and students’ perceived value of a subject is one factor influencing creativity (Amoah et al., 2015). Similarly, reflective practice involves the metacognitive ability to critically assess one’s experiences and actions. Students who value discipline may put more effort into supporting their behaviour and ability to act creatively (Mahmoodi et al., 2022). In the interim, reflective practice as a metacognitive behaviour may help foster personal development and creative thinking. Orakcı (2021) asserted that reflective behaviour differs across disciplines and that this difference is highly dependent on the nature of knowledge in each discipline. Thus, it is imperative to understand the values and interests the subject brings to students, as any educational endeavour is influenced by their perceived values and motivations (Liebendörfer and Schukajlow, 2020). Although research has explored the impact of task values on SRL strategies (Muwonge et al., 2019), few studies have examined the direct effect of students’ disciplinary values on reflective behaviour, particularly in creativity research.

*H2a:* Students’ disciplinary value significantly influences their reflective practice.

*H2b:* Reflective practice mediates the relationship between students’ disciplinary values and creativity in the learning process.

### Self-efficacy

In the model of human agency by Luszczynska and Schwarzer (2015), efficacy beliefs are directly related to individuals’ self-reflectiveness to realise a specific goal. Bartimote-Aufflick et al. (2016) explored the potential influence of self-efficacy on reflection by considering the four main steps: plan and work, initiate reflection, conduct a reflection session, and apply the outcome. Menon and Azam (2021) reported that teachers’ reflections on their past experiences and current teaching practices shaped their beliefs, emphasising the importance of fostering self-efficacy. Asakereh and Yousofi (2018) examined the relationships among reflective thinking, general self-efficacy, self-esteem, and academic achievement. Although self-efficacy often determines whether a person actively engages in creative activities, their explanation of differences in actual creative performance is relatively weak. From a creative learning perspective, people are confident in their creative abilities and value tasks (Haase et al., 2018; Pretz and McCollum, 2014). Reflective practice is a crucial aspect of creative potential and behaviour in learning. In addition, reflection involves risk, and individuals must be confident in their ability to recognise it as a growth opportunity (Ossa Parra et al., 2015).

Hence, higher self-efficacy is associated with greater activity, enhancing reflective practice related to creativity (Martin and Rimm-Kaufman, 2015). Harvey et al. (2016) suggest that reflective writing tasks offer opportunities to develop self-efficacy and motivation. Integrating the stated arguments makes a stronger sense of self-efficacy more likely to reflect positively on experiences. This link is consistent with the SRT, which describes how success in self-regulation (a positive correlation between creative SE and individual creativity) occurs through resource allocation during self-regulation.

*H3a:* Students’ self-efficacy significantly influences their reflective practice.

*H3b:* Reflective practice mediates the relationship between students’ self-efficacy and creativity in the learning process.

### Learning style

Tsingos et al. (2015) discussed differences in the use of learning styles and approaches, as well as the role of reflection. Learners with adaptive styles may feel more comfortable in social group meetings. Additionally, some studies provide a significant positive correlation between students’ learning methods and reflection (Tsingos-Lucas et al., 2016). Mahasneh et al. (2015) examined the relationship between learning styles (deep, surface, and strategic) and reflective thinking (habitual action, understanding, reflection, and critical reflection) among undergraduates. The literature shows that students’ skills, abilities, and engagement in reflective practice during the learning process are more closely linked to their learning styles (Alvi and Gillies, 2020). Moreover, learning styles and reflection are considered influential in the creative process in design education (Khaleghimoghaddam, 2023). One of the educational goals when working with design students is to teach them creative thinking and idea generation. Learning styles considerably impact how people generate ideas, reflect, and evaluate information. From this perspective, Al Maani (2022) investigated the relationships among reflective thinking, learning styles, and the degree of student progress in the architecture students’ design process and design products.

O’Connell et al. (2021) suggested that students who adopt in-depth learning approaches may exhibit greater creativity through reflective practice. Previous research on self-regulation has postulated the self-regulation and learning environment, academic achievement, differences in students’ learning outcomes due to self-regulation, and learning strategies (Galizty and Sutarni, 2021). From this perspective, the relationship between learning styles, reflection, and creativity is crucial. While empirical findings support the idea that understanding and incorporating learning styles into teaching can improve students’ creative insight, the mediating role of reflection has not received sufficient attention.

*H4a:* Students’ learning style significantly influences their reflective practice.

*H4b:* Reflective practice mediates the relationship between students’ learning styles and creativity in the learning process.

### Emotional management

Many empirical studies have examined how reciprocal interaction contributes to personal growth and resilience, as well as to improved decision-making (Namaziandost and Heydarnejad, 2023). Zee et al. (2016) emphasised the importance of emotional management in self-directed leadership development. The study revealed that leaders who effectively manage their emotions are more likely to engage in reflective practice. Emotional management enables leaders to receive and process feedback constructively, enhancing their ability to identify areas for improvement and take appropriate action. Emotional management and environmental, cognitive, and creative factors collectively shape an individual’s creative potential in multiple ways (Simsek and Serin, 2017). According to this view, the emotional dimension requires a mix of other factors to produce creative outcomes, and this mix varies across creative domains, tasks, or stages of the creative process. Verhaeghen (2021) found that reflection was critical in the relationship between emotional states (depressive symptoms) and creative performance. Similarly, Tong and Talwar (2022) found that resilience mediated the relationship between the two constructs and that gratitude moderated the indirect effect. Additional findings have been reported on humour, generosity, openness to experience, self-confidence, and mental resilience (Winton and Sabol, 2024).

Besides, reflective practice involves introspection and facilitates the examination of emotions and their impact on cognitive processes (Bassot, 2015). In the context of SRL, reflective behaviour can reconcile personal emotional factors and creative expression. Individuals engaged in reflective practice gain insight into their emotional responses, identify triggers for negative emotions, and develop strategies to manage them effectively (Hetland et al., 2013). By processing emotions through reflection, individuals can convert challenging experiences into valuable lessons and creative inspirations (González-Gómez and Richter, 2015).

*H5a:* Students’ emotional management significantly influences their reflective practice.

*H5b:* Reflective practice mediates the relationship between students’ emotional management and creativity in the learning process.

### Learning environment

An environment that encourages reflection provides a platform for self-expression and constructive feedback. Teachers organising the learning environment is one of the primary conditions affecting students’ enthusiasm (Macklem, 2015). Barrett et al. (2017) developed a learning environment that encourages autonomy and self-directed learning to explore how a student-driven art course affects reflective practice and creativity. Students can engage in deeper reflection through ongoing open projects, resulting in innovative and self-expressive works of art. In addition, a collaborative climate allows individuals to share their reflections and ideas, fostering diverse perspectives. The investigation highlights the influence of personal and environmental factors, confirming that administrators, time constraints, policies, and collaborative environments impact participants’ creative abilities (Griggs et al., 2018). Treacy and Gaunt (2021) explore the potential links between reflective practice, collective creativity, and collaborative learning in higher art education. While “learning environment” is not explicitly emphasised, their investigation into reimagining collective creativity resonates.

Pishghadam et al. (2016) indicated that students in an unsupportive environment were less likely to engage in reflective practice, resulting in limited creativity. Kim and Choi (2017) explored how a hostile learning climate influences students’ self-expression and creativity. While individual factors are internal processes, environmental factors influence and activate specific reflective behaviours, facilitating the process. Based on the literature, this study proposes a learning environment in which students can learn independently and receive feedback on their learning performance.

*H6:* The learning environment moderates the relationship between students’ reflective practice and creativity in the learning process.

### Reflective practice and creativity in the learning process

More recently, Lousberg et al. (2020) explored the role of reflection in architecture design education and its impact on students’ learning and development. The results showed a significant, but slight, increase in reflection during the design reflective practice. They concluded that students reflect at different levels when thinking about their work. However, for students to reach a higher level of reflection, supervisors must learn to reflect on themselves, as students’ reflective skills receive little attention in graduate training. Through reflection, students can critically analyse and evaluate their design work, identify areas for improvement, and explore alternative perspectives or solutions (Milovanovic et al., 2020). This process of critical self-reflection deepens their understanding of design and enables them to integrate new knowledge and insights from their experience.

The literature suggests that reflective practice is closely related to students’ understanding of complex design problems and their development of innovative solutions. It is the designer’s way of thinking about their previous experiences and includes covert design methods, with reflection at the centre of that process. Many scholars suggest that reflective practice is the learning goal or outcome of design pedagogy, since implicitness can be an essential technique for learning and design (Nurakenova and Nagymzhanova, 2024). Evaluating students’ reflective practice and learning environments is necessary to gain valuable insights and understand their cognitive behaviours related to the development of creativity (Kirkman and Brownhill, 2020).

*H7:* Reflective practice affects creativity in the learning process.

## Methods

In line with deductive logic, this study derived seven factors (personality characteristics, disciplinary value, self-efficacy, emotional management, learning style, reflective practice, and learning environment) from the literature that affected students’ creativity and proposed a conceptual framework. The survey method was employed to collect data regarding the research questions. A pilot survey was primarily used to help justify the instrument’s reliability and validity (Hossain, 2026). A questionnaire survey was used to gain an overall picture to answer the research questions. This section outlines the research design, justifies the research methods, and describes the specific steps in the research process. This study employed a stratified random sampling technique to select samples from three colleges and universities among the 54 target institutions. The colleges and universities were divided into three strata according to their classification. The samples were selected based on the proportions of each stratum within the total population (Hossain, 2025).

The questionnaire comprised three parts. The first part provided a brief introduction to the survey, helping participants quickly understand the background information. The second part focused on the general information of the respondents, including their gender, age group, and significant. The other third part was organised based on the measurements of the constructs from previous studies. Personality characteristics with 4 items were derived from Gosling et al. (2003), disciplinary value with 6 items was derived from Knekta et al. (2020), learning style with 5 items were derived from Wintergerst et al. (2003), emotional management with 5 items were derived from Wong and Law (2002), reflective practice with 6 items was derived from Sobral (2000), learning environment with 4 items was derived from Choi and Pak (2006), creativity in the learning process, with 5 items, was derived from Lin et al. (2014). The complete questionnaire, including measurement items, is administered on a five-point Likert scale. This study employs a cross-sectional research design to examine the correlated impact of SRL’s personal, behavioural, and environmental factors on undergraduate students’ creativity in learning.

### Data collection

Following comprehensive clarification of the research objectives and ethical commitments with the respondents, 714 online questionnaires were generated via the Tencent questionnaire platform and distributed via WeChat and the Tencent group for the respondents’ class. The formal data collection lasted six months, from December to April 2025. Five hundred twenty-three questionnaires (523) were gathered and meticulously recorded within the platform’s dataset. After duplicate entries and incomplete data were identified and removed, 402 datasets were deemed valid. Given the abundance of valid data exceeding the initial stratified sampling requirements, the present study uses the entire set for subsequent analyses. A PLS-SEM procedure was used to analyse the dataset.

A common variance testing method was used to assess the extent to which the data may be influenced by survey-related biases. Harman’s single-factor technique was employed to assess the extent of bias in the data (Kock, 2020). The Harman single-factor technique (Harman, 1967) uses exploratory factor analysis, loading all variables onto a single factor and constraining it, with no rotation (Podsakoff et al., 2003). The new factor is generally not in the researchers’ models; it is introduced for a specific analysis and then discarded. CMB may exist if the newly introduced common latent factor explains more than 50% of the variance. The principal component analysis results reveal that the data in our study are unaffected by common method bias, as the total variance extracted by a single factor is 22.374%, which is < 50%, the recommended threshold set by Harman (1967).

### Data analysis and findings

#### Demographic profile

The respondents in this study are final-year undergraduates from three colleges and universities in Shangdong province, mainland China, majoring in A&D (visual communication design, environment design, product design, and fashion design). Gender, age group, and academic level are considered when measuring respondents’ demographic variables, and descriptive and frequency statistics are used to analyse these variables. The results from the sample profile descriptive analysis show that there are no missing values or outliers in the statistics. Regarding gender, 60.7% (*n* = 244) of the respondents are female, and 39.3% (*n* = 158) are male. The respondents are also divided into five age groups: ≤18, 19–20, 21–22, 23–24, and ≥25. As indicated in the table, only 3.7% (*n* = 15) of the respondents are ≤18, 37.3% (*n* = 150) are 19–20, 5.5% (*n* = 22) are 23–24, 5.7% (*n* = 23) are ≥25, and 47.8% (*n* = 192) are 23–24, which is the highest percentage. Also presented in Table 1 are the participants’ academic activity, with the highest number of respondents (33.1%, *n* = 133) involved in visual communication design, followed by environment design (28.1%, *n* = 113), product design (20.4%, *n* = 82), and fashion design (18.4%, *n* = 74), the lowest percentage.

**Table 1:** Results of the path coefficient (*β*) analysis.

Hypotheses	Beta/OS	*T*	*P*	Significant
H1a: PC → RP	0.219	5.240	0.000	Yes
H2a: DV → RP	0.270	6.426	0.000	Yes
H3a: SE → RP	0.243	5.797	0.000	Yes
H4a: LS → RP	0.018	0.457	0.648	No
H5a: EM → RP	0.178	4.379	0.000	Yes
H6: RP → CILP	0.503	14.290	0.000	Yes

#### Evaluation of measurement model

**Figure 2:**
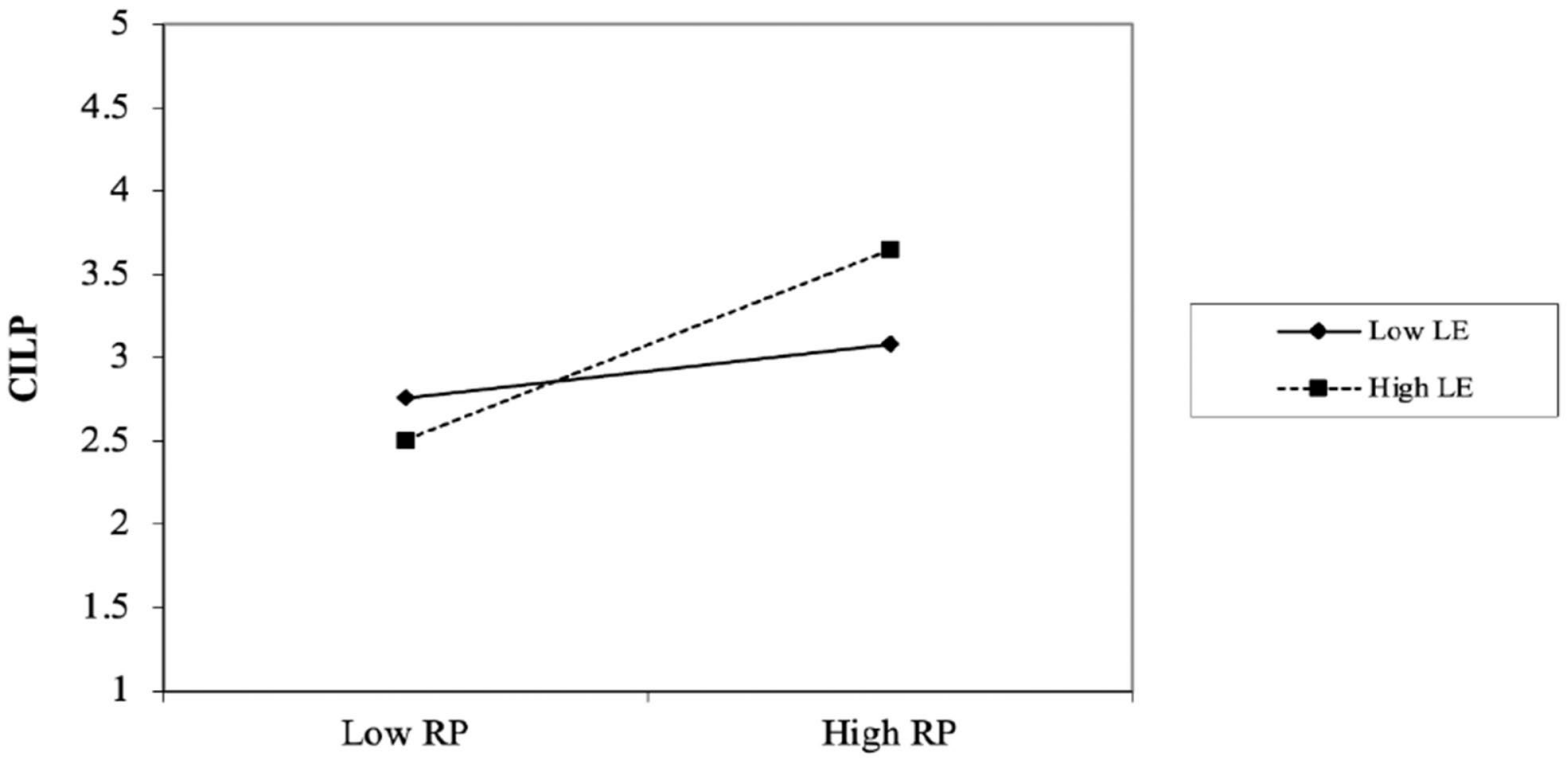
Results of the slope of the moderating effects analysis.

Initially, the outer loadings, CR, and *α* are assessed for construct reliability. Subsequently, convergent and discriminant validity are examined to ensure that the items converge with their constructs at the required level. The outer loadings (indicator reliability), α, and internal CR were examined to confirm construct reliability. Hair et al. (2014) opined that an outer loading of ≥0.7 can be deemed highly satisfactory. In this study, to meet the “highly satisfactory” criteria, all measurement items met the acceptable threshold, with values <0.7. As shown in Table 2 and Figure 2, following the second PLS operation, the outer loading ranged from 0.739 to 0.911, indicating that all indicators had individual reliability values ≥7.0 (Henseler et al., 2015). The reliability assessments for α and CR revealed that both exceeded the recommended value of 0.70 (Hair et al., 2017). As shown in Table 2, α is >0.70, and CR is >0.7, the cut-off value. The AVE approach is widely used to assess convergent validity. Table 2 shows that the average AVE for each latent variable in this study exceeds 0.5, indicating that each construct can explain more than half of the variance in its measured items (Hair et al., 2019). Convergent validity is confirmed.

**Table 2:** Assessment of reliability and convergent validity.

Constructs	Items	Loadings	Cronbach’s alpha	Composite reliability	AVE
Creativity in the learning process	CILP1	0.792	0.944	0.944	0.618
	CILP2	0.808			
	CILP3	0.777			
	CILP4	0.776			
	CILP5	0.773			
Disciplinary value	DV1	0.772	0.913	0.917	0.620
	DV2	0.786			
	DV3	0.763			
	DV4	0.774			
	DV5	0.789			
	DV6	0.810			
Emotional management	EM1	0.782	0.947	0.950	0.609
	EM2	0.775			
	EM3	0.764			
	EM4	0.739			
	EM5	0.808			
Learning environment	LE1	0.815	0.854	0.864	0.695
	LE2	0.829			
	LE3	0.857			
	LE4	0.832			
Learning style	LS1	0.893	0.980	0.992	0.802
	LS2	0.911			
	LS3	0.897			
	LS4	0.894			
	LS5	0.871			
Personality characteristics	PC1	0.813	0.931	0.942	0.617
	PC2	0.764			
	PC3	0.781			
	PC4	0.795			
	PC5	0.790			
Reflective practice	RP1	0.793	0.941	0.949	0.607
	RP2	0.771			
	RP3	0.768			
	RP4	0.798			
	RP5	0.759			
	RP6	0.784			
Self-efficacy	SE1	0.816	0.936	0.947	0.689
	SE2	0.837			
	SE3	0.835			
	SE4	0.833			
	SE5	0.812			
	SE6	0.830			

#### Discriminant validity assessment

The Fornell-Larcker approach suggests that the square root of each latent variable’s AVE should exceed its correlations with other variables (Fornell and Larcker, 1981). The output of the Fornell-Larcker approach is based on the square root of AVE in diagonals and the correlations below it (Ali et al., 2018). The formal criterion for interpreting the table instructs that if the top value, which is the square root of AVE in any column, is > than the values, which are the correlations below it, then discriminant validity exists. According to the Fornell–Larcker criterion results displayed in Table 3, the square root of each construct’s AVE is > than its correlation with other constructs, namely, CILP (0.786), DV (0.788), EM (0.780), LE (0.834), LS (0.896), PC (0.786), RP (0.779), SE (0.830). Hence, according to Hair et al. (2021), the data’s discriminant validity is acceptable.

**Table 3:** Results of the Fornell-Larcker criterion analysis to determine the discriminant validity.

Correlations	CILP	DV	EM	LE	LS	PC	RP	SE
CILP	**0.786**							
DV	0.200	**0.788**						
EM	0.282	0.150	**0.780**					
LE	0.123	0.030	0.048	**0.834**				
LS	0.147	0.138	0.154	−0.024	**0.896**			
PC	0.369	0.136	0.141	−0.004	0.144	**0.786**		
RP	0.505	0.365	0.287	0.087	0.150	0.321	**0.779**	
SE	0.227	0.148	0.147	0.008	0.148	0.152	0.345	**0.830**

PC, personality characteristics; DV, disciplinary value; SE, self-efficacy; LS, learning style; EM, emotional management; RP, reflective practice; CILP, creativity in the learning process.

### Assessment of structural model

In our study, the *R*^2^ analysis results, as displayed in Table 3, indicate that the R2 for the endogenous variable on creativity in the learning process is 0.292, indicating that the exogenous variables explain 29.2% of the variation, which is significant. The *R*^2^ for the reflective practice endogenous variable is 0.306, indicating that the exogenous variables explained 30.6% of the variation, which is also substantial. According to Hair et al. (2014) and Cohen (1988), F^2^ values of 0.02 ≤ F^2^ < 0.15, 0.15 ≤ F^2^ < 0.35, and F^2^ ≥ 0.35 denote small, medium, and large F^2,^ respectively. The findings portrayed in Table 3 reveal that the effect of DV (F^2^ = 0.099), EM (F^2^ = 0.043), PC (F^2^ = 0.065), and SE (F^2^ = 0.080) on RP meet the threshold of a small F^2^ (0.02 ≤ f^2^ < 0.15); while the effect of RP (F^2^ = 0.355) meets the threshold of a large F^2^ (F^2^ ≥ 0.35) effect on CILP. Also, the effect of PC, DV, SE, and EM on RP is small, while the effect of RP on CILP is large from a statistical and practical perspective. However, the effect of LS (F^2^ = 0.000) on RP is < the recommended value for small F^2^, indicating that LS does not influence RP. The relative impact of Q^2^ is assessed based on the cross-validated redundancy: 0.02 ≤ Q^2^ < 0.15 = weak; 0.15 ≤ Q^2^ < 0.35 = moderate; and Q^2^ ≥ 0.35 (Hair et al., 2019). In our study, a blindfolding test was performed to compute the Q2, which revealed that the entire model has a good fit and a medium Q^2^ (Table 4). The Q^2^ values for CILP and RP were 0.176 and 0.180, indicating that the endogenous latent variables are well reconstructed and that the model has moderate predictive relevance.

**Table 4:** Results of the coefficient of determination (*R*^2^), effect size (F^2^), and predictive relevance (Q^2^).

Variables name	Coefficient of determination	Predictive relevance	Effect size *f*^2^		
	*R* ^2^	Q^2^	Exogenous variables	RP	CILP
CILP	0.292	0.176	DV	0.099	
RP	0.306	0.180	EM	0.043	
			LS	0.000	
			PC	0.065	
			RP		0.355
			SE	0.080	

PC, personality characteristics; DV, disciplinary value; SE, self-efficacy; LS, learning style; EM, emotional management; RP, reflective practice; CILP, creativity in the learning process.

### Direct effects

This section presents the bootstrapping results for the direct effects of the independent and dependent variables (*β*). The *β*, *p*, *t*, and BC-CI values, based on 5,000 bootstrap resamples, are used to assess the significance of their *β*s. The statistical results confirmed that hypotheses H1a, H2a, H3a, H5a, and H6 are supported, whereas H4a is not. Table 1 shows the *β* results, which support five direct hypotheses. The supported hypotheses were significant, at least at the *p* ≤ 0.05 level, had expected sign directions, and had *β* coefficients ranging from 0.171 to 0.369.

Hypothesis (H1a) relates to PC and RP. The correlation was statistically significant as *p* < 0.05 (0.000) and *t* > 1.96 (5.240). Furthermore, the correlation was positive as *β* > 0.1 (0.219). Thus, PC significantly affects RP. Hypothesis (H2a) relates to DV and RP. The correlation was statistically significant as *p* < 0.05 (0.000) and *t* > 1.96 (6.426). Furthermore, the correlation was positive as *β* > 0.1 (0.270). Thus, DV significantly affects RP. Hypothesis (H3a) relates to SE and RP. The correlation was statistically significant as *p* < 0.05 (0.000) and *t* > 1.96 (5.904). Furthermore, the correlation was positive as *β* > 0.1 (0.243). Thus, SE significantly affects RP.

Hypothesis (H4a) relates to LS and RP. The correlation was statistically insignificant as *p* > 0.05 (0.648) and *t* < 1.96 (0.457). Furthermore, as *β* < 0.1 (0.018), the correlation was negative. Thus, LS does not significantly affect RP. Hypothesis (H5a) relates to EM and RP. The correlation was statistically significant as *p* < 0.05 (0.000) and *t* > 1.96 (4.379). Furthermore, the correlation was positive as *β* > 0.1 (0.178). Thus, EM significantly affects RP. Hypothesis (H6) relates to RP and CILP. The correlation was statistically significant as *p* < 0.05 (0.000) and *t* > 1.96 (14.290). Furthermore, the correlation was positive as *β* > 0.1 (0.503). Thus, RP significantly affects CILP.

### Indirect effects

Table 5 depicts the results of the potentially mediating effects of RP. The statistical results confirmed that hypotheses H1b, H2b, H3b, and H5b were statistically supported, but H4b was not. Hypothesis (H1b) relates to the mediating role of RP on the correlation between PC and CILP. Reflective practice (RP) had a significant indirect effect on the PC-CILP correlation. Hence, H1b was supported as *β* = 0.081, *t* = 4.477, *p* = 000, and 97.5% bias-corrected confidence interval (BC-CI) [LL = 0.048, UL = 0.119]. Hypothesis (H2b) relates to the mediating role of RP on the correlation between DV and CILP. Reflective practice (RP) had a significant indirect effect on the DV-CILP correlation. Hence, H2b was supported as *β* = 0.099, *t* = 5.000, *p* < 0.05, and 97.5% BC-CI [LL = 0.063, UL = 0.142]. Hypothesis (H3b) relates to the mediating role of RP on the correlation between SE and CILP. Reflective practice (RP) had a significant indirect effect on the SE-CILP correlation. Hence, H3b was supported as *β* = 0.090, *t* = 4.549, *p* < 0.05, and 97.5% BC-CI [LL = 0.056, UL = 0.136]. Hypothesis (H4b) relates to the mediating role of RP on the correlation between LS and CILP. Reflective practice (RP) had an insignificant indirect effect on the LS-CILP correlation. Hence, H4b was not supported as *β* = 0.005, *t* = 0.306, *p* = 0.759, and 95% BC-CI [LL = −0.025, UL = 0.033]. Hypothesis (H5b) relates to the mediating role of RP on the correlation between EM and CILP. Reflective practice (RP) had a significant indirect effect on the EM-CILP correlation. Hence, H5b was supported as *β* = 0.073, *t* = 3.281, *p* < 0.05, and 97.5% BC-CI [LL = 0.031, UL = 0.117].

**Table 5:** Results of the indirect mediating effects.

Indirect effect	Beta/OS	95% confidence interval bias corrected		*T*	*P*	Supported
		LL	UL			
H6 PC → RP → CILP	0.081	0.048	0.119	4.477	0.000	Yes
H7 DV → RP → CILP	0.099	0.063	0.142	5.000	0.000	Yes
H8 SE → RP → CILP	0.090	0.056	0.136	4.549	0.000	Yes
H9 LS → RP → CILP	0.005	−0.025	0.033	0.306	0.759	No
H10 EM → RP → CILP	0.063	0.044	0.128	3.772	0.000	Yes

OS, original sample; LL, lower limit; UL, upper limit; *T*, *T* statistics; *P*, *P* value; * *p* < 0.05.

As shown in Table 6, the bootstrapping results indicate that the moderating effect of LE is statistically significant (*t* = 4.293, *β* = 0.206, *p* = 0.000, *p* < 0.05). Moreover, the BC-CI [LL = 0.118, UL = 0.297] does not straddle a 0. Additionally, without considering the moderating effect (RP*LE), the *R*^2^ for CILP was 0.356. This indicates that RP accounts for 35.6% of the change in CILP. However, including the interaction term (RP*LE) increased *R*^2^ to 0.369, demonstrating that RP*LE accounts for 1.3% of the variance in the dependent variable (CILP). This indicates that increased LE strengthens the relationship between RP and CILP.

**Table 6:** Results of the moderating effects.

Hypothesis 7	Beta/OS	95% confidence interval bias corrected		*T*	*P*	*F* ^2^	Supported
		LL	UL				
LE*RP → CILP	0.206	0.118	0.297	4.293	0.000	0.014	Yes

LE, learning environment; RP, reflective practice; CILP, creativity in the learning process.

As shown in Figure 2, the line is much steeper for high LE (the dashed line), indicating that at a high LE level, the impact of RP on CILP is much stronger than at a low LE level. However, at a low LE level (solid line), the line tends to flatten, indicating that at lower LE levels, the increase in RP does not produce a similar change in the CILP. Thus, it can be surmised that a higher LE level intensifies the impact of RP on CILP.

## Discussion

The statistical findings confirmed that students’ characteristics have a positive, significant effect on their reflective practice. This result aligns with recent studies examining how personality shapes individuals’ reflective tendencies and practices (Brown and Slater, 2023). Using a Big Five framework, Marashi and Rezaei (2023) confirmed that presentational characteristics (neuroticism, openness, and conscientiousness) affect teaching reflections. These positive and significant findings align with the idea that individuals with certain personality traits are more inclined to engage in reflective activities, which, in an educational context, can lead to more meaningful learning experiences (Kaufman et al., 2016). Moreover, the findings indicate that students’ personality characteristics affect their creativity during learning through reflective practice, suggesting that personality traits can enhance students’ creative abilities when reflective behaviour is highly engaged. The findings confirmed that reflective practice was a significant mediator between personality traits and creativity in students’ learning process, in line with Hong and Choi (2019).

This finding supported the idea that students who attach a high value to a task will employ more complex cognitive and metacognitive strategies. Instilling interest and value in an academic setting can better mobilise students’ cognitive engagement in higher-level strategies and reflection (Stajkovic and Sergent, 2019). Moreover, the findings confirmed that reflective practice was a significant mediator between the disciplinary value and creativity in the learning process. It was consistent with the idea that critical reflection and assessment can deepen a student’s understanding of the subject, thereby developing creative thinking skills aligned with learning goals. This finding was consistent with previous studies, which showed that individuals’ efficacy beliefs are directly related to their self-reflectiveness. The findings suggest that students are driven by confidence in their abilities, taking a more active and thoughtful approach to reflection and deeper assessment. In line with previous studies by Hill et al. (2008), this finding supports the notion that individuals with stronger self-efficacy are more likely to reflect positively on their learning experiences, thereby enhancing personal creativity. Individuals must have confidence in their ability to reflect to achieve better growth. Therefore, the higher the self-efficacy, the more positive the reflective practice. This mediating effect of reflective practice highlights the importance of developing reflective skills in educational settings as they bridge the gap between self-efficacy beliefs and creative outcomes.

This finding was consistent with some recent Grobbel (2013) research and failed to identify a significant relationship between learning style and reflective practice. The consistent results challenged the idea that students’ skills, abilities, and engagement in reflective practice are based on their learning style (Alvi and Gillies, 2020; van Diggele et al., 2020). According to the findings, reflective practice did not mediate the relationship between learning style and creativity during learning. This finding could be attributed to the complexity of these structures, the possibility of independent effects, unexplored mediating factors, or limitations in reflective measures. In the realm of Chinese design education, characterised by highly regulated curricula and pedagogy, individual learning style preferences may be stifled or overshadowed by standardised instructional approaches. The conceptual alignment between LS and RP may be tenuous in this specific sample owing to cultural, disciplinary, or developmental influences. This non-significant pathway provides a valuable theoretical insight rather than indicating a fault in the model. It questions the presumed universality of learning style theories and emphasises the need to contextualise their explanatory value within specific educational frameworks.

The above findings on emotional management are consistent with those of Namaziandost and Heydarnejad (2023), who found a positive correlation between emotion regulation and RP, suggesting that teachers who manage their emotions are more likely to engage in active, critical self-reflection when responding to classroom challenges effectively. Furthermore, this finding was consistent with a few studies that reported a mediating role for reflective practice in influencing emotional management and creativity. For example, González-Gómez and Richter (2015) suggested that positive, activated, and promotion-focused emotional states are particularly likely to encourage creative ideas when reflective practice is highly recommended. Du et al. (2021) found that negative emotions can positively affect creativity through self-reflection.

The statistical findings revealed that reflective practice had a positive, significant impact on creativity during the learning process. It suggested that A&D students who could reflect on their learning experiences showed higher levels of creative learning capability. The finding confirmed the theoretical foundations laid on the significance of reflection in the learning process and the role of reflective practice in the design discipline, emphasising its contribution to creativity. This was consistent with the growing body of literature emphasising the positive impact of reflective practice on student creativity in various design domains. The statistical findings showed that the learning environment significantly moderated the reflective practice and creativity in the learning process. The literature increasingly recognises the importance of the learning environment in shaping students’ reflective practice and creative development. This finding aligns with the notion that students’ reflective behaviour regarding creativity is closely linked to the learning environment. For instance, found that self-directed learning and an open classroom environment can encourage students to engage in reflective dialogue and self-assessment, leading to deeper reflection and innovative, self-expressive works of art.

### Theoretical implications

This study contributes to a deeper understanding of the factors that influence the learning and creativity of Chinese A&D students. First, this study incorporated personal, behavioural, and environmental factors of SRL into creativity research, enriching the creativity literature and shedding light on the potential relevance and applicability of SRL theory in A&D education. The conceptual research framework provided a structured, comprehensive lens for investigating the dynamics of creativity in the learning process. Second, this study contributes to a better understanding of the determinants and multifaceted nature of creativity in A&D education. It highlighted the importance of personal factors, including personality traits, disciplinary values, self-efficacy, and emotion regulation, as well as the role of reflective practice. It sheds light on the significance of individual characteristics in the creative process. More importantly, it confirmed the theoretical underpinnings proposed in previous studies by demonstrating that those factors significantly influence reflective practice. This reaffirmed the relevance of personal factors in the context of SRL and their role in motivating self-regulation behaviours (Grobbel et al., 2013).

Moreover, contrary to some studies that prioritised learning styles as the primary determinants of learning outcomes, this study found no significant relationship between learning styles and RP, or between RP and its subsequent effect on creativity. This highlighted the need for a more nuanced understanding of how learning styles may or may not influence creative outcomes. Thirdly, this research advanced existing knowledge on the impact of metacognitive behaviour on individual development and creativity. More specifically, by exploring the mediating role of reflective practice, this study highlighted the significance of reflective behaviour in A&D students’ learning, reinforcing the notion that self-awareness, reflection, and thoughtful engagement in the learning process are crucial for nurturing creativity. Moreover, the findings on the moderating role of the learning environment highlighted its importance, not just as an auxiliary or external factor, but as a dynamic component that actively shapes the relationship between reflective practice and creativity. The results suggested that while reflective practice is an integral part of the creative process, its effectiveness depends on the quality and nature of the learning environment. In a favourable learning environment, the impact of reflective practice on creativity may be amplified. In contrast, in an unconducive environment, the benefits of reflective practice may be suppressed or even negated.

Taken together, this study highlighted those areas that had been overlooked in previous research, particularly the role of metacognitive behaviour and individual engagement, stressing the need for a more comprehensive understanding of the drivers of creativity in the context of A&D. It emphasised the complexity of creativity and the interplay between the cognitive, behavioural, emotional, and motivational aspects of learning. This contrasts with a more monolithic approach, arguing that multiple aspects of an individual’s learning process must be addressed to foster creativity. In addition, given China’s unique cultural and educational environment, these findings provide deeper insights into the traits that foster creativity in this context. They hint at the underlying cultural factors that influence creativity and its determinants.

### Practical implications

The theoretical insights from this study offer valuable practical implications for educators, policymakers, institutions, and researchers within A&D education. By translating the theoretical findings into actionable strategies, stakeholders can enhance students’ self-regulation and creative learning experiences and better align educational outcomes with industry expectations. First, student-centred A&D education is premised on recognising individual differences, emphasising personal stances, and attaching importance to behavioural attributes. Educators should respect students’ personality development and foster their sense of self-efficacy. Educators also need to help students understand the multifaceted nature of creativity and give appropriate feedback to help improve their metacognition and recognise their creative strengths and limitations.

Second, policymakers should consider implementing curriculum reforms, enhancing teacher training, and revising evaluation standards. These can include incorporating SRL-related skills into professional curricula and equipping teachers with the knowledge and skills to guide students in SRL, for example, by training teachers to use interventions (such as developing students’ self-reflective skills, journaling, and writing) to guide students in developing effective learning strategies that can create opportunities and challenges for creative thinking. Third, institutions should actively use research findings to optimise the learning environment, ensuring that they foster creativity, promote SRL, and respond to students’ diverse needs. They also need to ensure that the school climate encourages student autonomy, collaboration, reflection, and adaptability, keeping pace with the country’s cultural and social development needs for creative talent. Therefore, colleges and universities should seek opportunities to collaborate with industry to help students gain real-world experience that meets industry expectations.

Additionally, researchers should expand their work by exploring the interactions between SRL and creativity across diverse cultural and educational contexts. In summary, the practical implications call for a shift in focus from a one-size-fits-all approach to a more personalised, reflective, and industry-aligned educational experience. By actively shaping the learning environment and nurturing SRL practices, educational institutions can foster creativity among A&D students and equip them with the skills needed to meet evolving demands.

### Limitations and future research

Acknowledging research limitations clarifies the context of the research findings and provides a pathway for future research. First, students self-reported their learning creativity using a Likert scale. The method evaluates learners’ subjective creative participation but not design outputs, problem-solving, or domain-specific competencies. Without artefact-based evaluation, expert opinion, peer review, or organised pedagogical feedback, the creativity outcomes and processes of design education lack construct validity. Overgeneralizing regarding creative development may result from self-perceived appraisals that bias responses and overestimate relationships. Creative learning engagement may be better described than design creativity in its usual disciplinary sense. This reduces the validity of the findings in real-world design education. The Consensual Assessment Technique (CAT) and Creative Product Semantic Scale (CPSS) can better measure creativity in design projects, portfolios, and prototypes. The theoretical framework and literature review on the growth of Chinese art and design students’ creativity are based on Western ideas of creativity and learning. Limitations on Chinese educational, cultural, and institutional scholarship are contextual. The study does not critically analyse how sociocultural norms, pedagogical traditions, and the structural characteristics of Chinese art and design education affect students’ creative learning processes, or how the cultural transferability and contextual boundaries of acquired theories are shaped. Chinese art and design education creative research should apply context-sensitive, culturally based theories.

Second, there was a limitation regarding the population. This study focused on a sample of final-year A&D undergraduates from three universities in Shandong province, China. Future research should incorporate diverse student populations to enhance the generalisability of the findings, for example, by comparing first-year and final-year students, or by comparing students from different design domains, and by including more provinces or conducting comparative studies between Hong Kong, Taiwan, Macao, and mainland China. Finally, the research methodology limitations. This study primarily employed a quantitative methodology based on self-reported measures. Future research could benefit from a more balanced mix of quantitative and qualitative methods to better understand the phenomenon.

## Data Availability

The raw data supporting the conclusions of this article will be made available by the authors, without undue reservation.
