# Correction: Strive for excellence: unboxing the antecedents of art design students' creativity in the learning process in China

**DOI:** 10.3389/fpsyg.2026.1831016

**Published:** 2026-04-07

**Authors:** Jin Jianhao, Veronica Ng, Lim Chong Hin, Mohamed Rizal Mohamed, Md Sazzad Hossain

**Affiliations:** 1 School of Fine Arts and Design, Heze University, Heze, China; 2 School of Architecture, Building and Design, Taylor's University, Subang Jaya, Malaysia; 3 Faculty of Arts and Social Sciences, Sunway University, Subang Jaya, Malaysia; 4 Faculty of Built Environment, Universiti Teknologi MARA, Shah Alam, Malaysia; 5 College of Tourism and Hospitality, University of Tabuk, Tabuk, Saudi Arabia

**Keywords:** creativity in the learning process, disciplinary value, emotional management, learning environment, learning style, personality characteristics, reflective practice, self-efficacy

Affiliation Faculty of Built Environment, Universiti Teknologi MARA, Shah Alam, Malaysia was erroneously given as School of Education, Universiti Teknologi MARA, Shah Alam, Malaysia.

The original version of this article has been updated.

